# Effect of reverse transcriptase inhibitors on LINE-1 and Ty1 reverse transcriptase activities and on LINE-1 retrotransposition

**DOI:** 10.1186/1471-2091-12-18

**Published:** 2011-05-05

**Authors:** Lixin Dai, Qing Huang, Jef D Boeke

**Affiliations:** 1 Department of Molecular Biology and Genetics, Johns Hopkins University School of Medicine, Baltimore, MD 21205, USA

## Abstract

**Background:**

LINE-1s (L1, Long Interspersed Element-1) are the most abundant autonomous non-LTR retrotransposons in the human genome and replicate by reverse transcription of an RNA intermediate. Full-length L1 encodes two open reading frames (ORF1, ORF2) and ORF2 has reverse transcriptase activity.

**Results:**

Here we expressed human L1 RT in *E. coli *and the purified protein displayed the same RT activity as that of ORF2p expressed in insect cells. We tested the effect of different reverse transcriptase inhibitors on L1 RT and found that all four tested nucleoside inhibitors efficiently inhibited L1 RT activity competitively. The K_i _values of NRTIs were calculated (AZTTP, 16.4 ± 4.21 nM; d4TTP, 0.73 ± 0.22 nM; ddCTP, 0.72 ± 0.16 nM; 3TCTP, 12.9 ± 2.07 nM). L1 RT was less sensitive to non-nucleoside reverse transcriptase inhibitors, among these nevirapine had no effect, even at concentrations up to 500 μM. We also examined the effect of RT inhibitors on L1 retrotransposition efficiency *in vivo *using a cell-based retrotransposition assay. Similarly, all analog inhibitors decreased L1 retrotransposition frequency with different potencies whereas nevirapine had little or no effect on L1 retrotransposition. For comparison, we also tested the same inhibitors to highly purified RT of an LTR-retrotransposon (Ty1) and found it was less sensitive to NRTIs than L1 RT and has the same inhibition profile as L1 RT to NNRTIs.

**Conclusions:**

These data indicate that bacterially expressed L1 RT is an active reverse transcriptase sensitive to nucleoside RT inhibitors but not to non-nucleoside inhibitors.

## Background

Long interspersed element-1s (L1 or LINE-1) are non-LTR (Long Terminal Repeat) retrotransposons accounting for ~17% of human DNA [1]. Though most L1 copies are functionally inactive, there are ~80-100 retrotransposition-competent L1s in human genome [2]. L1s have greatly shaped the human genome by their own retrotransposition and mobilization of non-autonomous elements (*Alu*, SINEs) *in trans *[3-6]. A full-length L1 element is about 6 kb in length and contains a 5' untranslated region (UTR), two non-overlapping open reading frames (ORF1 and ORF2), followed by a short 3' UTR that ends in a poly adenosine tail [7-12]. The product of ORF1 encodes a 40 kDa protein (ORF1p) with nucleic acid binding and chaperone activities [13-16]. ORF2 encodes a ~150 kDa multifunctional protein (ORF2p) with endonuclease (EN) [17], reverse transcriptase (RT) activities [18-20] and a cysteine-rich domain of unknown function [21]. The life cycle of L1 begins with the transcription of the L1 mRNA, which is exported to the cytoplasm for translation. L1 proteins have a strong *cis*-preference and are proposed to specifically associate with their encoding mRNAs to form an RNP particle that re-enters the nucleus and integrates into the genome [22-24]. Several lines of evidence suggest that L1 transposes via a mechanism known as target primed reverse transcription (TPRT) [25], in which reverse transcription of L1 RNA is the crucial step. Results from a cell-based retrotransposition assay indicate that L1 retrotransposition depends on active RT function [25,26]. Full-length human ORF2 protein expressed in baculovirus-infected insect cells has strong RNA-dependent and DNA-dependent DNA polymerase activities [19,20,25]. Until now, active L1 RT or ORF2p had not been successfully expressed in prokaryotic hosts such as *E. coli*.

It is known that reverse transcriptases are susceptible to RT inhibitors classified into three types: nucleoside analog inhibitors (NRTIs), nucleotide analog inhibitors (NtRTIs), and non-nucleoside inhibitors (NNRTIs). The first two groups of inhibitors are structural analogs of natural deoxynucleotides and, upon phosphorylation to the triphosphate form in the cell, compete with dNTPs for access to the active site of reverse transcriptase. Since all analog inhibitors lack a 3'-hydroxyl group, they act as DNA chain terminators and generally have a broad spectrum of inhibitory activity [27]. The NNRTIs, on the other hand, are structurally diverse hydrophobic chemicals that function in a distinct manner. Instead of being incorporated into the nascent DNA strand, they specifically bind to a "NNRTI pocket" motif formed by the HIV-1 RT p66 subunit [28,29]. Binding of NNRTIs to this motif distorts the nearby HIV-1 RT catalytic site and thus blocks DNA synthesis. All NNRTIs specifically inhibit HIV-1 RT activity non-competitively without themselves being structurally modified in the cell.

Recent publications have indicated that two NNRTIs (nevirapine and efavirenz) effectively reduce cell proliferation and promote cell differentiation by inhibiting endogenous RT activity [30-32]. They were also found to inhibit the growth of human tumors in animal models. To explain these phenomena, it was hypothesized that endogenous RTs might be involved in a mechanism controlling cell proliferation and differentiation. As the most abundant source of endogenous RTs, L1 was assumed to be the major target of these RT inhibitors [31]. Separately, previous studies have indicated that nucleoside analog RT inhibitors (but not NNRTIs) could suppress L1 retrotransposition activity in a tissue culture assay [33,34].

To directly characterize the susceptibility of L1 RT to various RT inhibitors, we overexpressed and purified recombinant human L1 reverse transcriptase in *E. coli*. Then we tested the effect of NRTIs and NNRTIs on L1 RT directly by a cell-free RT assay. The K_i _values of four NRTIs against L1 RT were determined. We also investigated the effect of these drugs on L1 retrotransposition frequency using a cell-based retrotransposition assay. The data from both cell free and tissue culture experiments demonstrated correlated results: all NRTIs inhibited L1 RT activity and retrotransposition efficiency, whereas nevirapine had no significant effect on L1 RT in either type of assay.

## Results

### Protein expression and *in vitro *RT activity

**Figure 1:**
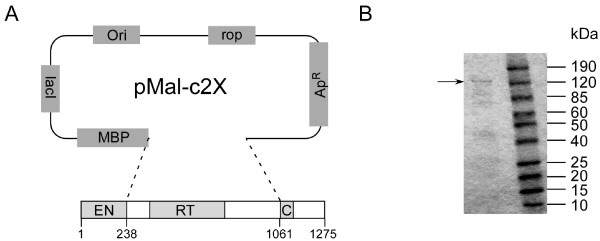
**Expression of human L1 reverse transcriptase in *E. coli***. (A) Domain structure of human L1 ORF2 protein. The conserved domains are shown as shaded rectangles. L1 RT domain (Aa 238-1061) was cloned into pMal-c2X downstream of MBP gene in frame. (B) Human L1 RT domain was expressed in *E. coli *as a fusion protein (MBP-RT) and purified as described in materials and methods. The sample was resolved by 4-20% SDS/PAGE with Coomassie blue staining. Protein molecular mass markers are to the right (kDa). The arrow indicates the mobility of MBP-RT fusion protein.

Human L1 ORF2 encodes 1275 amino acids and contains three functional domains. Among them, the structure of EN domain (amino acids 1-239) has been determined [17,35]. The RT domain spans ~1/3 (amino acids ~380-773) of the ORF2 sequence and the cysteine-rich domain starts at amino acid ~1130 (Figure 1a). We cloned the RT domain from a synthetic human L1 element - *ORFeus*-Hs [36], which encodes the same amino acid sequence as native L1_RP_, with the linker regions extended to the boundaries of the EN and C domains (amino acids 238-1061) into pMal-c2x vector (Figure 1A) and expressed L1 RT as a fusion protein with an MBP tag located at the N-terminus. The protein had the expected size of ~140 kDa on SDS-PAGE (Figure 1B). Strong RT activity was detected in both the crude cell lysate and purified protein by homo-polymer RT assays. Meanwhile, a control lysate prepared from cells transformed with empty pMal-c2x vector and purified MBP-ORF1 protein did not show any RT activity, indicating that the RT activity was L1 RT-derived rather than from host cell components or the MBP tag. One unit of RT activity was defined as the amount of enzyme necessary to catalyze incorporation of 1 nmol dTTP into poly (rA)-oligo (dT)_12-18 _in 30 min at 37°C. The specific activity of purified L1 RT was calculated as 0.375 unit per μg protein (poly (rA)-oligo (dT)_12-18 _primer/template) and 0.094 unit per μg protein (poly (rI)-oligo (dC)_12-18 _primer/template).

### Effect of RT inhibitors on activities of different reverse transcriptases

**Figure 2:**
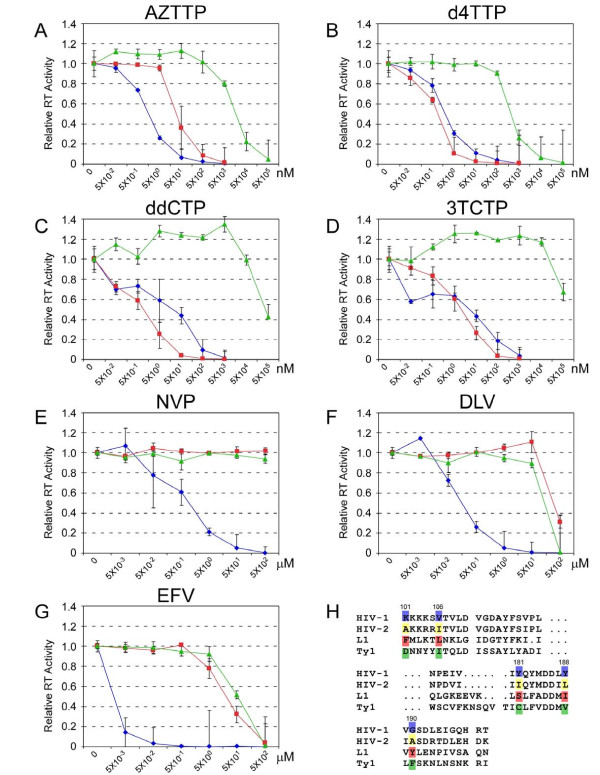
**Effect of RT inhibitors on the activities of HIV-1, L1 and Ty1 RTs**. The reverse transcriptase activities of HIV-1 RT (blue diamond), L1 RT (red square) and Ty1 RT (green triangle) were measured as described in the materials and methods. The RT activity of control assay without inhibitors was considered as 1.0. The activity in the presence of inhibitors was indicated as relative activity with respect to the control. All experiments were done at least three times and standard deviations are shown. (A) AZTTP. (B) d4TTP. (C) ddCTP. (D) 3TCTP. (E) NVP. (F) DLV. (G) EFV. (H) Alignment of partial sequences of HIV-1 p66, HIV-2, L1 and Ty1 RTs. Residues (101, 106, 181, 188, 190) important to NNRTI pocket motif are highlighted. Sequence from 122-174 is not shown.

We tested the effect of four NRTIs and three NNRTIs on HIV-1, L1 and Ty1 reverse transcriptase activities. As shown in Figure 2A, B, C and 2D, all four triphosphate NRTIs markedly inhibited the activities of HIV-1 and L1 RTs at nM concentrations. Conversely, NRTIs inhibited Ty1 RT activity at the μM level, if at all. We also found that Ty1 RT was more sensitive to the thymine analogs than to cytidine analogs. Interestingly, inhibitors AZTTP, ddCTP and 3TCTP modestly but significantly increased Ty1 RT activity (up to 40%) at low concentrations.

NNRTIs were previously reported to inhibit HIV-1 RT activity with different potencies (NVP, K_i _= 200 nM; EFV, K_i _= 2.93 nM; DLV, IC_50 _= 260 nM) [29,37,38] and we observed similar degrees of inhibitory effects on HIV-1 RT here (Figure 2E, F, G). In contrast, L1 and Ty1 RTs were inhibited only in the presence of high concentrations of DLV (50 μM) and EFV (500 μM). NVP showed no inhibition at concentrations as high as 500 μM. Amino acids K101, V106, Y181, Y188 and G190 in the HIV-1 RT p66 subunit are believed to be important for binding NNRTIs [39]. We aligned the sequences of HIV-1, HIV-2, L1 and Ty1 RTs (Figure 2H) and found the sequences of HIV-2, L1 and Ty1 RTs at above positions were all different from HIV-1 RT, suggesting that the "NNRTI pocket" is absent from these RTs.

### Kinetics of inhibition of L1 RT by NRTIs

**Figure 3:**
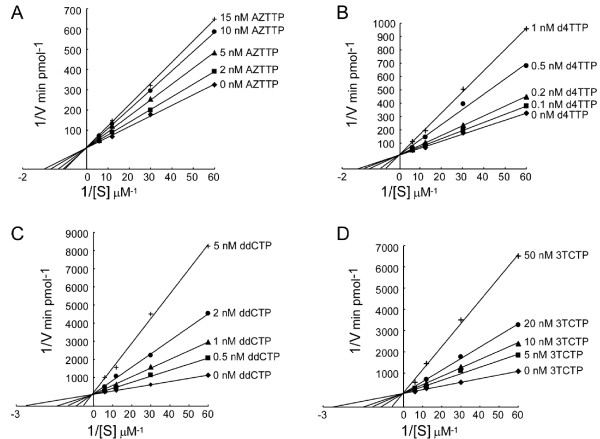
**Kinetic analysis of inhibition of L1 RT by NRTIs**. The L1 RT activity was measured as described in materials and methods. (A) Double reciprocal plot of the velocity of the L1 RT activity as a function of [^32^P]-dTTP substrate concentration. Increasing concentrations of substrate in the absence (diamond) or presence of 2 nM (square), 5 nM (triangle), 10 nM (circle) or 15 nM (+) AZTTP. (B) Double reciprocal plot of the velocity of the L1 RT activity as a function of [^32^P] dTTP substrate concentration. Increasing concentrations of substrate in the absence (diamond) or presence of 0.1 nM (square), 0.2 nM (triangle), 0.5 nM (circle) or 1 nM (+) d4TTP. (C) Double reciprocal plot of the velocity of the L1 RT activity as a function of [^32^P] dCTP substrate concentration. Increasing concentrations of substrate in the absence (diamond) or presence of 0.5 nM (square), 1 nM (triangle), 2 nM (circle) or 5 nM (+) ddCTP. (D) Double reciprocal plot of the velocity of the L1 RT activity as a function of [^32^P] dTTP substrate concentration. Increasing concentrations of substrate in the absence (diamond) or presence of 5 nM (square), 10 nM (triangle), 20 nM (circle) or 50 nM (+) 3TCTP.

Having determined that all NRTIs inhibited L1 RT activity, we next studied the kinetics of inhibition of L1 RT by these NRTIs. Poly (rA)-oligo (dT)_12-18 _and [^32^P]-dTTP were used as template/primers and substrate to assay thymine analogs (AZTTP, d4TTP). Poly (rI)-oligo (dC)_12-18 _and [^32^P]-dCTP were used to assay cytidine analogs (ddCTP, 3TCTP). The RT assays were repeated with the same amount of template/primer in the presence of different amount of substrates and inhibitors. The results were analyzed by double-reciprocal (Lineweaver-Burk) plots. As expected, inhibition of L1 RT with respect to both dTTP and dCTP substrates showed classic competitive behavior with no significant change of the V_max _(Figure 3). The K_m _value for dTTP was 0.83 μM and the apparent K_m _values in the presence of 2 nM, 5 nM, 10 nM and 15 nM AZTTP were 0.96 μM, 1.15 μM, 1.27 μM and 1.42 μM respectively. The K_i _value for AZTTP against L1 RT was calculated as 16.4 ± 4.21 nM (Table 1). The apparent K_m _values in the presence of 0.1 nM, 0.2 nM, 0.5 nM and 1 nM d4TTP were 0.95 μM, 0.99 μM, 1.58 μM and 2.15 μM and the K_i _value for d4TTP was 0.73 ± 0.22 nM. Of the cytosine analogs, the K_m _value for dCTP substrate was 0.38 μM and the apparent K_m _values in the presence of 0.5 nM, 1 nM, 2 nM and 5 nM ddCTP were 0.76 μM, 0.86 μM, 1.34 μM and 2.62 μM. The apparent K_m _values in the presence of 5 nM, 10 nM, 20 nM and 50 nM 3TCTP were 0.45 μM, 0.69 μM, 0.93 μM and 2.17 μM. The K_i _values calculated for ddCTP and 3TCTP were 0.72 ± 0.16 nM and 12.9 ± 2.07 nM respectively (Table 1). Since all NRTIs are competitive inhibitors against L1 RT with respect to their corresponding natural dNTPs, the IC_50 _value can be calculated as IC_50 _= K_i_/(1+ S/K_m_). The IC_50 _values of AZTTP, d4TTP, ddCTP and 3TCTP were calculated as 19.8 nM, 0.88 nM, 1.04 nM and 18.6 nM.

**Table 1:** Kinetic constants for the inhibition of L1 RT by NRTIs

RT inhibitors	AZTTP	d4TTP	ddCTP	3TCTP
K_i _(nM)	16.4 ± 4.21	0.73 ± 0.22	0.72 ± 0.16	12.9 ± 0.16

### Effect of RT inhibitors on L1 retrotransposition

**Figure 4:**
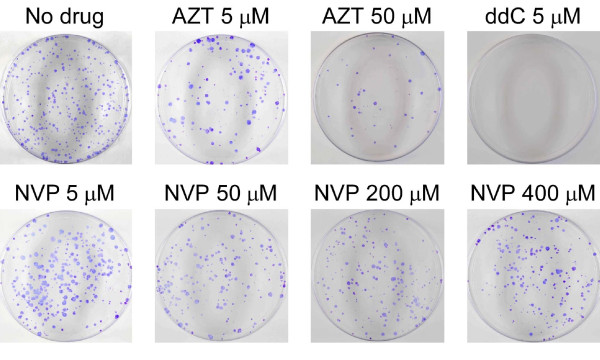
**Effect of RT inhibitors on *ORFeus*-Hs retrotransposition efficiency**. A tissue culture cell-based L1 retrotransposition assay was done as described in materials and methods. Example plates from retrotransposition assay indicating different effects of RT inhibitors on L1 retrotransposition. See Table 2 for details.

To further evaluate the effect of RT inhibitors on L1 *in vivo*, we tested L1 retrotransposition in the presence of these chemicals using an established tissue culture retrotransposition assay. All transposition assays were performed using *ORFeus*-Hs that encodes wild type ORF1 and ORF2 proteins but transposes at a 2~3 fold higher frequency (~2%) than the corresponding native human L1 [36]. As shown in Figure 4 and Table 2, the RT inhibitors had diverse effects on human L1 retrotransposition. All seven NRTIs decreased L1 retrotransposition with various potencies. AZT and d4T reduced L1 retrotransposition efficiency to similar levels when 5 μM inhibitors were added to the medium, but d4T had a stronger effect at higher concentrations (50 μM) (Table 2). The inhibitors 3TC, ddC, and ddI strongly reduced L1 retrotransposition at a low concentration (5 μM) and the last two completely abolished L1's transposition ability. The only NtRTI we tested, bis-POM PMPA, also reduced the retrotransposition rate to almost zero at 5 μM. NNRTIs DLV and EFV inhibited L1 retrotransposition efficiency by ~72% at concentrations of 50 μM and 5 μM respectively. But nevirapine only decreased L1 transposition frequency by ~10% at all tested concentrations (Figure 4, Table 2). Retrotransposition assays at higher concentrations of DLV and EFV could not be carried out due to high levels of seemingly nonspecific cell death caused by these drugs.

**Table 2:** Effect of reverse transcriptase inhibitors on *ORFeus*-Hs retrotransposition.

	RT inhibitors	Relative retrotransposition efficiency^a^			
		5 μM	50 μM	200 μM	400 μM
		
NRTI	AZT	0.62 ± 0.06	0.23 ± 0.04		
	d4T^b^	0.50 ± 0.05	0.05 ± 0.04		
	ddC	<0.005^c^	<0.005^c^		
	3TC	0.08 ± 0.03	0.03 ± 0.03		
	ddI	<0.005^c^	<0.005^c^		
	ABC	0.74 ± 0.06	0.20 ± 0.04		
	FTC	0.25 ± 0.01	0.12 ± 0.04		
					
NtRTI	Bis-POM PMPA^b^	<0.005^c^	<0.005^c^		
					
NNRTI	NVP^b^	1.08 ± 0.13	0.89 ± 0.03	0.89 ± 0.00	0.87 ± 0.03
	DLV^b^	0.63 ± 0.00	0.28 ± 0.03		
	EFV^b^	0.28 ± 0.02	- ^d^		

a Transposition efficiency of control assay without inhibitors was considered as 1.0. Transposition efficiency in the presence of inhibitors was indicated as relative efficiency with respect to the control. All data were normalized to cell viability with the treatment of the same amount of inhibitors. Data are mean of six independent experiments ± standard error

b Transposition efficiency was compared to the control containing the same amount of DMSO

c No colonies when 1×10^4 ^cells were plated

d Transposition efficiency could not be determined due to excessive cell death

## Discussion

Biochemical dissection of the L1 retrotransposition mechanism necessitates *in vitro *expression of L1 encoded ORFs. Thus far, only the function of the EN domain of ORF2 has been clearly demonstrated *in vitro *[35], leaving the RT and cysteine-rich domains less well understood. Full-length L1 ORF2 protein was previously expressed in eukaryotic hosts such as yeast and insect cells with relatively low yield and purity [19,40]. We describe a fragment of the L1 RT expressed in bacterial cells as a highly active polymerase fusion protein. Ability to detect robust activity suggests that post-translational modification by eukaryotic host is not critical to L1 RT activity. Given the efficiency and simplicity of *E. coli *expression system, this will allow extensive follow-up biochemical studies of the L1 retrotransposition mechanism. Recombinant L1 RT expressed in *E. coli *displayed strong reverse transcriptase activity with both homopolyer substrates tested though higher activity was observed with poly (rA)-oligo (dT)_12-18 _as previously seen with L1 ORF2p from insect cells [19]. Furthermore, the specific activity of L1 RT is essentially the same as that of full-length ORF2p expressed in insect cells [19] although the bacterially expressed fusion protein studied here lacks the EN and cysteine-rich domains. Interestingly, L1 ORF2p expressed in yeast lost 84% RT activity when amino acids 1-161 were deleted and 50% of activity when amino acids 952-1275 were removed [40], suggesting the importance of these two regions to its RT activity. Similar requirement was reported for recombinant Ty1 RT, which required C-terminal residues of integrase for its RT activity [41]. It should be noted that the previously reported L1 ORF2p (yeast) RT activity was measured in the context of a cell-free lysate and at an extremely low level. Our findings obtained from highly active and purified protein indicate that EN and cysteine-rich domains are dispensable for RT function although we cannot exclude the possibility that the N-terminal MBP tag may act as a substitute for one of the two domains, helping the RT fold into active conformation. Indeed, we were not successful at retaining RT activity following proteolytic removal of the MBP tag, consistent with its importance in solubility and/or activity of the protein.

Expression of the highly active L1 RT allows *in vitro *biochemical comparisons of reverse transcriptases from phylogenetically related non-LTR retrotransposons and LTR-retrotransposons. Direct comparison under the same assay condition revealed that L1 and HIV-1 RTs are distinct from one another and have distinct interaction patterns with RT inhibitors. Both RTs were markedly inhibited by NRTIs and displayed similar susceptibilities to d4TTP and 3TCTP. Compared to HIV-1 RT, L1 RT was less sensitive to AZTTP but more sensitive to ddCTP. Obvious differences were observed for NNRTIs that inhibited HIV-1 RT activity, as expected [29,38,42] but had little effect on L1 RT. It is notable that the recombinant Ty1 RT was less susceptible to all RT inhibitors tested in this study. The recombinant Ty1 RT used here is essentially the same as that used in a previous publication [41] but its specific activity with poly (rA)-oligo (dT)_12-18 _and poly (rI)-oligo (dC)_12-18 _template/primer is several times higher than that using poly (rC)-oligo (dG)_12-18 _(data not shown) [41]. Previous studies have indicated that the guanosine analog ddGTP decreases Ty1 RT activity by 90% when the ratio of ddGTP/GTP reaches 0.2 [41,43]. In this work Ty1 RT activity was inhibited only when the concentrations of inhibitors were several thousand times higher than the native substrate. It is known that reverse transcriptases react differently to inhibitors using different types of template/primer. Perhaps the resistance of Ty1 RT to inhibitors is due to the different effects of template/oligo mixes, while it is also possible that adenosine and cytidine analogs are not recognized efficiently by recombinant Ty1 RT.

The RT inhibitor AZT was reported to suppress L1 retrotransposition in the cell-based retrotranspostion assay [34,44]. In this study we have illustrated the effect of eleven different RT inhibitors on L1 retrotransposition frequency, which is correlated with data obtained from *in vitro *RT assay. For example, AZT with a higher K_i _inhibited the L1 retrotransposition less proficiently than d4T with a lower K_i _value, and the same pattern was observed for cytidine analogs. Both thymine analogs are relatively weak inhibitors of L1 retrotransposition compared to HIV-1, whose production is completely inhibited by 1-3 μM AZT [45]. Cytidine analogs (ddC, 3TC) and ddI, on the other hand, have strong inhibitory effects comparable to those on HIV. This significant difference may be caused by the metabolism of these inhibitors *in vivo *since the triphosphate form of 3TC and ddC were found to be ten times of that of AZT in the cell [46]. We have also tested these RT inhibitors on the retrotransposition of a highly active synthetic mouse L1 (*ORFeus-Mm*) [47,48]. The obtained results show the same inhibition profile observed with *ORFeus-Hs*: all NRTIs inhibit L1 retrotransposition, yet NVP doesn't inhibit *ORFeus-Mm *retrotransposition profoundly, even at high concentrations (Table 3). Though synthetic LINE-1 elements (*ORFeus*) were used in this study, they all encode the same amino acid sequences as wild-type L1s and their retrotransposition is fully dependent on RT function.

**Table 3:** Effect of reverse transcriptase inhibitors on *ORFeus-Mm *retrotransposition.

	RT inhibitors	Relative retrotransposition efficiency^a^			
		**5 μM**	**50 μM**	**200 μM**	**400 μM**
		
NRTI	AZT	0.75 ± 0.08	0.16 ± 0.02		
	d4T^b^	0.28 ± 0.05	0.01 ± 0.02		
	ddC	<0.0025^c^	<0.0025^c^		
	3TC	0.21 ± 0.07	0.08 ± 0.01		
	ddI	<0.0025^c^	<0.0025^c^		
	ABC	0.80 ± 0.05	0.15 ± 0.03		
	FTC	0.34 ± 0.01	0.26 ± 0.04		
					
NtRTI	Bis-POM PMPA^b^	<0.0025^c^	<0.0025^c^		
					
NNRTI	NVP^b^	0.72 ± 0.05	0.66 ± 0.06	1.12 ± 0.08	0.79 ± 0.05
	DLV^b^	0.60 ± 0.05	0.02 ± 0.01		

a Transposition efficiency of control assay without inhibitors was considered as 1.0. Transposition efficiency in the presence of inhibitors was indicated as relative efficiency with respect to the control. All data were normalized to cell viability with the treatment of the same amount of inhibitors. Data are mean of six independent experiments ± standard error.

b Transposition efficiency was compared to the control containing the same amount of DMSO

c No colonies when 1×10^4 ^cells were plated

d Transposition efficiency could not be detected due to excessive cell death

Phylogenetic evidence also suggests that non-analog inhibitors are specific to HIV-1 RT and ineffective against other polymerases. Sequence alignment of HIV-1 p66, HIV-2, L1 and Ty1 RTs indicates the positions important for NNRTI binding are highly variable. These substitutions change not only the conformation but also the electronic charge of the NNRTI binding motif, such as the replacement of lysine (basic) by phenylalanine (acidic, aromatic) by L1 at position 101. It is known that HIV-2 is resistant to NNRTIs because of the destruction of "NNRTI pocket" motif [39]. Various studies have indicated that substitutions at positions 103, 106, 181, 188 and 190 are the most common HIV-1 mutants that reduce NVP susceptibility more than 50 fold [49]. In addition, NVP and EFV are inactive against a variety of polymerases including AMV RT, MLV RT, human DNA polymerases α, β, γ and Klenow fragment [29,38]. HIV-1 mutants resistant to EFV normally have substitutions at positions 101 and 103 suggesting EFV may contact a smaller surface of the RT [38]. This may explain our observation that EFV still inhibits L1 RT activity but with lower effectiveness. Taken together, our results above suggest that nevirapine is specific to HIV-1 RT and does not inhibit L1 RT activity both *in vitro *and *in vivo*. Given this, we conclude that the reported anti-tumor function of NVP and EFV is due to another mechanism distinct from inhibition of endogenous L1 RT activity. An alternative anti-tumor mechanism may result from the cytotoxicity of RT inhibitors since EFV and DLV were found to cause massive HeLa cell death at concentrations of 50 μM and 100 μM respectively.

## Conclusion

In summary, we report the expression and purification of recombinant human L1 RT in bacterial host cells for the first time. The protein has the same reverse transcriptase activity as the full-length ORF2 expressed from insect cells suggesting no host specific modifications are required for RT activity. We have tested the effect of different RT inhibitors against L1 RT activity and retrotransposition by *in vitro *(cell-free) and *in vivo *(tissue culture) analyses. The data presented here indicated that L1 RT is sensitive to NRTIs but NNRTIs inhibit L1 RT less efficiently. Nevirapine, an RT inhibitor with reported anti-tumor function, has no effect on L1 RT activity.

## Methods

### Abbreviations

3TC, 2',3'-dideoxy-3'-thiacytidine; 3TCTP, 2',3'-dideoxy-3'-thiacytidine triphosphate; ABC, [(1*R*)-4-[2-amino-6-(cyclopropylamino)purin-9-yl]-1-cyclopent-2-enyl]methanol; AZT, azidothymidine; AZTTP, azidothymidine triphosphate; bis-POM PMPA, 2-(6-aminopurin-9-yl)ethoxymethylphosphonic acid; d4T, 2'-3'-didehydro-2'-3'-dideoxythymidine; d4TTP, 2'-3'-didehydro-2'-3'-dideoxythymidine triphosphate; ddC, 2'-3'-dideoxycytidine; ddCTP, 2'-3'-dideoxycytidine triphosphate; ddI, 2'-3'-dideoxyinosine; DLV, delavirdine; FTC, 4-amino-5-fluoro-1- [2-(hydroxymethyl)- 1,3-oxathiolan-5-yl]- pyrimidin-2-one; EFV, efavirenz; NVP, nevirapine; NRTI, Nucleoside Reverse Transcriptase Inhibitor; NNRTI, Non-nucleoside Reverse Transcriptase Inhibitor; NtRTI, Nucleotide Reverse Transcriptase Inhibitors (NtRTIs); RT, reverse transcriptase. TPRT, Target Primed Reverse Transcription.

### HIV-1 RT and RT inhibitors

Recombinant HIV-1_BH10 _RT p66 produced from *E. coli *was obtained from University of Alabama at Birmingham, Center for AIDS Research, Gene Expression core Facility through the NIH AIDS Research and Reference Reagent Program, Division of AIDS, NIAID, NIH. AZTTP, d4TTP, ddCTP and 3TCTP were purchased from ChemCyte Inc (San Diego, CA, USA). AZT, d4T, ddC, 3TC, ddI, ABC, FTC and bis-POM PMPA were purchased from Sigma-Aldrich (St. Louis, MO, USA). NVP, DLV and EFV were purchased from Research Toronto Chemicals (North York, ON, Canada). Template/primer mix poly (rA)-oligo (dT)_12-18_, poly (rI) and oligo (dC)_12-18 _were purchased from Midland Certified Reagent Company (Midland, TX, USA). [α-^32^P] dTTP and [α-^32^P] dCTP were purchased from PerkinElmer Life And Analytical Sciences, Inc. (Waltham, MA, USA). Transfection reagent Fugene 6 was purchased from Roche Applied Science (Indianapolis, IN, USA). All RT inhibitors were first made as 100 mM stock solutions. d4T, bis-POM PMPA, NVP, DLV, EFV were dissolved in pure DMSO and other chemicals were dissolved in water. Template/primer mix poly (rI)-oligo (dC)_12-18 _was made by mixing poly (rI) and oligo (dC)_12-18 _according to Cheng *et al *[50].

### Plasmids

Synthetic human L1 ORF2 sequences were created by following a codon optimization procedure as previously described [47], and were ligated into pBluescript KS(-) (Stratagene, Santa Clara, CA, USA) to make pWA112.

To make pWA195, pCEP puro was first made by replacing the hygromycin resistance cassette pCEP4 (Invitrogen, Carlsbad, CA) with a 1.7 kb Sal I fragment of puromycin resistance cassette from pPGKpuro (a gift from Dr. Peter Laird). Plasmid pCEPpurosmL1 was made by replacing the pCEP4 backbone with pCEPpuro using *Not*I/*Bam*HI sites. Plasmid pWA195 was made by replacing smL1 in pCEPpurosmL1 with the synthetic human L1 coding sequence from pBSshL1.

Plasmid pLD48 was constructed by PCR amplification of pWA112 with the primers JB11578 (5'-CCGGATCCCGCATCAAGAACCTGACCCAGAGCC-3') and JB11584 (5'-ACGCGTCGACTTAGTAGATCTGCTTCAGCTCGTTGTAG-3'), digestion of the product with *Bam*HI and *Sal*I, and cloning of the product into *Bam*H I and *Sal *I sites of pMal-c2x (NEB, Ipswich, MA, USA). Human L1 ORF2 amino acid 238-1061 was inserted behind MBP gene in frame, and a "*TAA*" stop codon was introduced at the end of the insert. Plasmid pMal-ORF1 was constructed by cloning full-length human L1 ORF1 into the *Bam*HI and *Sal*I sites of pMal-c2x plasmid. Both constructs were confirmed by sequencing.

pQH1 was constructed by subcloning a DNA fragment containing the last 115 amino acids of Ty1 integrase and full-length RT-RH from pGEX-4T-3 [51] into the *Nde*I and *Pst*I sites of the pCold I plasmid (Takara, Japan).

### Expression and purification of human L1 and Ty1 RTs

MBP tagged L1 RT was overexpressed and purified according to the protocol provided by NEB. Plasmid pLD48 was transformed into *E. coli *BL21 cell and plated on LB with 100 μg/ml carbenicillin. 200 ml LB medium (0.2% glucose, 100 μg/ml carbenicillin) was inoculated with a 2 ml overnight culture and grown at 37°C with shaking to an OD_600 _of 0.3, followed by induction with 0.5 mM IPTG for 3 h at 37°C. Cells were harvested by centrifugation at 4000 × g for 20 minutes and the supernatant was discarded. The cells were resuspended in 5 ml column buffer (20 mM Tris-Cl pH 7.4, 200 mM NaCl, 1 mM EDTA) and lysed on ice with sonication with five bursts of 15 sec. The lysate was centrifuged at 9,000 × g for 30 mins and the supernatant was loaded onto 1 ml amylose resin, which was washed with 10 ml column buffer and then the fusion protein was eluted with column buffer with 10 mM maltose. The majority of protein eluted in the first two fractions, which were pooled and concentrated using a Centricon YM-100. Glycerol was added to a final concentration of 50% and enzyme was stored at -20°C.

pQH1 was co-transformed with chaperone plasmid pG-Tf2 (Takara, Japan) into *E. coli *BL21 cell. Cells were grown at 37°C in LB medium containing 50 μg/ml of carbenicillin, 34 μg/ml chloramphenicol and 1 ng/ml tetracycline until *A*_600 _reached 0.4. After 30 minutes incubation at 15°C, expression of recombinant Ty1 RT was induced with 0.5 mM IPTG. The cells were further cultured for 24 hours at 15°C, harvested by centrifugation and stored at -80°C. Recombinant Ty1 RT was sequentially purified on a HisTrap chelating column, a desalting column, a cation exchange column, a Superdex 200 gel filtration column, and again on a HisTrap chelating column with an Äkta FPLC system (GE Healthcare, Piscataway, NJ, USA). Eluted protein from the last step of purification was dialyzed overnight into the dialysis buffer (20 mM HEPES-NaOH pH 7.5, 300 mM NaCl, 10% glycerol, 1 mM EDTA, and 0.1 mM TCEP), concentrated to 6 mg/ml as measured by Bio-Rad Bradford assay, and stored at -80°C in 50 μl aliquots.

### *In vitro *RT assay

The RT assays for HIV-1 and L1 RT were performed in a 20 μl reaction mixtures containing 50 mM Tris-Cl (pH 8.0), 50 mM KCl, 5 mM MgCl_2_, 10 mM DDT, 0.01 U template/primer, 1 μl [α-^32^P] dTTP or [α-^32^P] dCTP (final concentration 0.17 μM, 3000 Ci/mmol, 10 mCi/ml) at 37°C for 30 min. Then the mixture was spotted on DE81 paper and washed three times with 2×SSC buffer for a total time of 30 min. The DE81 paper was dried and counted by scintillation counter Beckman LS6000SC. RT assay for Ty1 RT was done under the same buffer condition except that 20 mM MgCl_2 _was used and the reaction mixture was incubated at room temperature. In each reaction the amounts of L1, HIV-1 and Ty1 RTs with the same specific activity were added. To test the inhibition of the RT inhibitors, 1 μl inhibitor was included in the mixture to obtain the desired concentration. In testing the effect of NVP, DLV and EFV, 1 μl pure DMSO was added in the positive control reaction. All assays were done at least in triplicate.

### Kinetic analysis of NRTIs

The kinetic analysis was performed under the same conditions described above but with various concentrations of substrates and inhibitors as indicated in the text. Poly (rA)-oligo (dT)_12-18 _and [α-^32^P] dTTP were used to analyze AZTTP and d4TTP. Poly (rI)-oligo (dC)_12-18 _and [α-^32^P] dCTP were used to assay ddCTP and 3TCTP.

### Cell culture and L1 retrotransposition assay

The cell-based retrotransposition assay was conducted as described [26]. HeLa cells (a gift from Dr. John Moran, University of Michigan) were seeded in 6-well dishes in DMEM (2×10^5 ^cells/well). The next day cells were transfected with Fugene 6 according to the manufacturer's manual. Each transfection consisted of 96 μl Opti-Mem, 1 μg pWA195 DNA and 3 μl Fugene 6. Sixteen hours after transfection, the cells were trypsinized and transferred to a 6 cm plate in DMEM with 2.5 μg/ml puromycin and the RT inhibitors. Three days after puromycin selection, dead cells were removed and puromycin resistant cells were trypsinized and counted with a hemocytometer. The puromycin resistant cells were plated on a 10 cm plate (1×10^4 ^cells/plate) in 10 ml DMEM with 500 μg/ml G418. The RT inhibitors were added to the same concentration as in the puromycin selection. For assays of d4T, bis-POM PMPA, NVP, EFV and DLV, the same amount of DMSO (final conc <0.01%) was added to the control plate. Two weeks later, G418 resistant cells were fixed to the plate and stained with 0.1% crystal violet. The number of G418 resistant colonies was counted to calculate retrotransposition frequency. Six independent assays were done for each RT inhibitor.

### Cell cytotoxicity of RT inhibitors

Untransfected Hela cells were plated on 10 cm plate (500 cells/plate) with and without RT inhibitors. Ten days later, cells were fixed to the plate and stained with 0.1% crystal violet. The number of colonies was counted to calculate colony formation ability. Colony formation ability of control assay without inhibitors was considered as 1.0 and colony formation ability in the presence of inhibitors was indicated as relative efficiency with respect to the control.

### Sequence alignment

The RT sequences of HIV-1, HIV-2, L1 and Ty1 RTs were aligned automatically by Clustal X [52] and manually adjusted according to Shaharabany *et al *[53] and Wilhelm *et al *[41].

## Competing interests

The authors declare that they have no competing interests.

## Authors' contributions

LD and JDB designed the experiments; LD and QH performed the experiments. LD and JDB wrote the manuscript. All authors read and approved the final manuscript.
